# PLEKHA5 regulates the survival and peritoneal dissemination of diffuse-type gastric carcinoma cells with Met gene amplification

**DOI:** 10.1038/s41389-021-00314-1

**Published:** 2021-03-06

**Authors:** Yuko Nagamura, Makoto Miyazaki, Yoshiko Nagano, Masako Yuki, Kiyoko Fukami, Kazuyoshi Yanagihara, Kazuki Sasaki, Ryuichi Sakai, Hideki Yamaguchi

**Affiliations:** 1 Department of Cancer Cell Research, Sasaki Institute, Sasaki Foundation, Tokyo, Japan; 2 Laboratory of Genome and Biosignal, School of Life Sciences, Tokyo University of Pharmacy and Life Sciences, Tokyo, Japan; 3 Division of Biomarker Discovery, Exploratory Oncology Research and Clinical Trial Center, National Cancer Center, Chiba, Japan; 4 Department of Peptidomics, Sasaki Institute, Sasaki Foundation, Tokyo, Japan; 5 Department of Biochemistry, Kitasato University School of Medicine, Kanagawa, Japan

**Keywords:** Oncogenes, Growth factor signalling

## Abstract

Met gene amplification has been found in a subset of malignant carcinomas, including diffuse-type gastric carcinoma (DGC), which has a poor prognosis owing to rapid infiltrative invasion and frequent peritoneal dissemination. Met is considered a promising therapeutic target for DGC. However, DGC cells with Met gene amplification eventually acquire resistance to Met inhibitors. Therefore, identification of alternate targets that mediate Met signaling and confer malignant phenotypes is critical. In this study, we conducted a phosphoproteomic analysis of DGC cells possessing Met gene amplification and identified Pleckstrin Homology Domain Containing A5 (PLEKHA5) as a protein that is tyrosine-phosphorylated downstream of Met. Knockdown of PLEKHA5 selectively suppressed the growth of DGC cells with Met gene amplification by inducing apoptosis, even though they had acquired resistance to Met inhibitors. Moreover, PLEKHA5 silencing abrogated the malignant phenotypes of Met-addicted DGC cells, including peritoneal dissemination in vivo. Mechanistically, PLEKHA5 knockdown dysregulates glycolytic metabolism, leading to activation of the JNK pathway that promotes apoptosis. These results indicate that PLEKHA5 is a novel downstream effector of amplified Met and is required for the malignant progression of Met-addicted DGC.

## Introduction

Gastric cancer is one of the leading causes of cancer-associated mortality worldwide. There are two major histological subtypes of gastric adenocarcinomas according to the Laurén classification: intestinal and diffuse^1^. Diffuse-type gastric carcinoma (DGC) consists of poorly differentiated carcinoma cells and often exhibits aggressive progression. DGC is characterized by rapid infiltrative growth accompanied with massive stromal fibrosis and frequent metastasis to the peritoneum^2,3^. These aggressive characteristics contribute to the poor prognosis of patients with DGC^4,5^. Several genetic alterations have been implicated in DGC. Germline mutations in *CDH1*, which encodes E-cadherin, result in hereditary DGCs^6^. Genome sequencing of gastric carcinoma has shown that highly recurrent mutations of *RHOA* occur exclusively in DGC^7–9^. Gene amplification of *MET* and *FGFR2* has also been observed in DGC^10–12^.

The oncogene *MET* encodes Met receptor-type tyrosine kinase, whose ligand is hepatocyte growth factor (HGF). Met signaling regulates multiple aspects of cancer malignancies, including cell migration and invasion, cell proliferation and survival, and angiogenesis^13^. Met is aberrantly activated by point mutations, gene amplification, overexpression, gene fusion, and alternative splicing in a small but significant fraction of various cancer types^14,15^. In addition, Met gene amplification is a major cause of resistance to EGF receptor tyrosine kinase inhibitors in non-small cell lung cancer^16^. Therefore, Met is considered a promising therapeutic target, and dozens of Met inhibitors have been evaluated in clinical trials^17^.

Met gene amplification is correlated with poor prognosis in patients with gastric cancer^12,18,19^. We and other groups have reported that gastric cancer cell lines exhibiting Met amplification are addicted to Met signaling and are highly sensitive to Met inhibitors^19–21^. These findings provide a rationale for the use of Met inhibitors to treat gastric cancers with Met gene amplification. However, the use of tyrosine kinase inhibitors eventually causes drug resistance, which is also observed with Met inhibitors. Several studies have shown that carcinoma cells with Met gene amplification acquire resistance to Met inhibitors both in vitro and in vivo^22,23^. Thus, it is essential to elucidate the mechanism of Met-inhibitor resistance and to identify alternate molecular targets downstream of Met signaling.

In a previous study, we identified Met as a major tyrosine-phosphorylated protein in DGC cells and revealed that Met is required for the growth and peritoneal dissemination of DGC cells with Met gene amplification^21^. In this study, we systematically identified the tyrosine-phosphorylated proteins in Met-addicted DGC cells. Among them, we found that a protein called Pleckstrin Homology Domain Containing A5 (PLEKHA5) is a critical regulator of malignant phenotypes, including peritoneal dissemination, of DGC cells addicted to Met signaling.

## Materials and methods

### Cell culture

Human cell lines used in this study were listed in Supplementary Table 1. These cells were maintained in RPMI 1640 medium (Sigma-Aldrich) supplemented with 10% fetal bovine serum (FBS) and antibiotics at 37 °C in a humidified atmosphere containing 5% CO_2_. Met-inhibitor-resistant 58As9 cells were established by culturing 58As9 cells in the continuous presence of 300 nM PHA-665752 or JNJ-38877605 for 3–6 weeks. For deprivation analysis, cells were cultured in DMEM (Sigma) with or without glucose or pyruvate, supplemented with 10% dialyzed FBS. Mycoplasma contamination was tested using a MycoAlert Mycoplasma Detection Kit (Lonza).

### Reagents and antibodies

Commercially available antibodies used in this study were listed in Supplementary Table 2. A polyclonal anti-PLEKHA5 antibody was generated as described previously^24^. PHA-665752, crizotinib (PF-2341066), saracatinib (AZD0530), AG1478, and JNJ-38877605 were purchased from Selleck Chemicals. Anisomycin and 2-deoxyglucose were purchased from Wako Chemicals. Doxorubicin and Nutlin-3 were purchased from Sigma-Aldrich and Cayman Chemical, respectively.

### Affinity purification and identification of tyrosine-phosphorylated proteins

Tyrosine-phosphorylated proteins were affinity-purified from 58As9 cells as described previously^21^. The purified proteins were subjected to SDS-PAGE, stained using a Silver Stain MS Kit (Wako), excised, digested with trypsin, and subjected to liquid chromatography-tandem mass spectrometry analysis. The proteins were identified using a Mascot MS/MS ion search.

### siRNA transfection

Stealth RNAi molecules against Met (#1, HSS106478; #2, HS106479), PLEKHA5 (#1, HSS122935; #2, HSS122936), PLEKHA6 (#1, HSS117794; #2, HSS1176991), and the negative control (12935-300) were purchased from ThermoFisher Scientific. Cells were transfected with the indicated siRNAs using Lipofectamine RNAiMAX Reagent (ThermoFisher Scientific). The transfected cells were cultured for 24–72 h and then used for immunoblotting and other assays. Cell migration and invasion assays were performed 24–48 h after siRNA transfection. For the cell growth assay, cells were transfected with siRNA again 3 days after the first transfection and were cultured for another 3 days before the assay.

### DNA microarray analysis

Total RNA was isolated from 58As9 cells transfected with the control or PLEKHA5 siRNA using the RNeasy kit (Qiagen). The Clariom D assay (Affymetrix) was used for gene expression profiling. Microarray hybridization, washing, staining, and scanning were performed according to the standard Affymetrix protocols. Array data export, processing, and analysis were performed using the Affymetrix GeneChip Command Console Software. All the microarray datasets have been deposited in GEO (accession number, GSE163063). Only genes encoding proteins and upregulated or downregulated at least twofold in PLEKHA5 knockdown versus control cells were subjected to further analyses. Complete lists of the genes are available in Supplementary Table 3. Gene enrichment analysis was conducted using the Metascape webpage (https:// www.metascape.org) and the data are available in Supplementary Tables 4 and 5.

### Immunoblotting and immunoprecipitation

Cells were lysed in a buffer containing 50 mM HEPES–NaOH (pH7.0), 150 mM NaCl, 10% glycerol, 1% Triton X-100, 1.5 mM MgCl_2_, 1 mM EGTA, 1 mM Na_3_VO_4_, 100 mM NaF, and a protease inhibitor cocktail (Roche). The samples were subjected to immunoblot analysis as described previously^21^. For immunoprecipitation, 2–6 µg of antibodies were incubated with 0.5–1 mg of cell lysates for 2 h, and then with protein G magnetic beads (MBL) for 1 h at 4 °C. In some cases, antibodies were cross-linked to the protein G magnetic beads according to the manufacturer’s protocols. Immunoprecipitates were washed with lysis buffer and then subjected to immunoblotting.

### Cell proliferation assay

Cells were plated onto 96-well plates at 1–2 × 10^3^ cells/well and cultured for 3–6 days in the presence or absence of siRNAs or inhibitors. Cell growth was determined using a Premix WST-1 Cell Proliferation Assay System (Takara) or Cell Counting Kit-8 (Dojindo).

### Apoptosis assay

DNA fragmentation associated with apoptotic cell death was measured using a Cell Death Detection ELISA plus kit (Roche). To observe apoptotic cells by live-cell imaging, cells were transfected with siRNAs for 2 days and then stained with IncuCyte NucLight Red BacMam 3.0 (Essen Bioscience) and IncuCyte Annexin V Green Reagent for apoptosis (Essen Bioscience). Phase-contrast and fluorescence images of the cells were captured with the 10× objective every 1 h for 3 days using an IncuCyte live-cell analysis system (Essen Bioscience).

### Cell cycle analysis

Cells were transfected with siRNAs and stained with 2 µg/ml of propidium iodide in PBS containing 0.5 µg/ml of RNase A. The DNA content of cells was determined using BD FACS Aria II (BD Bioscience). The obtained data were analyzed using FlowJo software ver. 10 (FlowJo).

### Cell migration and invasion assays

Cell migration and invasion assays were performed with FluoroBlok 24-multiwell insert systems (8 μm pore size, Corning). For migration assay, the lower surface of the inserts was coated with 50 µg/ml of collagen type-I (Corning) for 1 h at room temperature. For invasion assay, the lower surface of the inserts was coated with 100 µg/ml Matrigel (Corning) for 1 h at room temperature and then 50 µl of 330 µg/ml Matrigel was added to the inserts and solidified for 1 h at 37 °C. 58As9 cells were fluorescently labeled with 20 µM Calcein-AM (Dojindo) for 30 min at 37 °C. The labeled cells (4 × 10^4^ for migration or 1 × 10^5^ for invasion) were suspended in 300 µl of serum-free medium and added to the upper chambers. A growth medium (800 μl) containing 10% FBS was added to the lower chambers as a chemoattractant. After 8 h incubation for migration or 24 h for an invasion at 37 °C, cells that migrated onto the lower surface of the inserts were directly imaged by fluorescent microscopy and were counted in 5 randomly selected fields per insert.

### Lentiviral transduction

Lentiviral particles were produced by transfecting 293FT cells (ThermoFisher Scientific) using ViraPower (ThermoFisher Scientific) and lentiviral constructs, pLKO.1-puro (a generous gift from Bob Weinberg^25^, Addgene plasmid #8453) containing control shRNA (CCTAAGGTTAAGTCGCCCTCGCTCGAGCGAGGGCGACTTAACCTTAGG) or PLEKHA5 shRNA (CCAAGGATGACTGTGGAAGAGCTCGAGCTCTTCCACA-GTCATCCTTGG). Two to three days after transfection, the cell supernatant containing lentiviral particles was collected. The 58As9 cells were infected with the lentiviruses for 1 day and then subjected to intraperitoneal injection into nude mice 2 days after infection.

### Peritoneal dissemination assay

BALB/c nude mice (6-week-old, female) purchased from CLEA Japan were inoculated intraperitoneally with 58As9 cells (2 × 10^6^). Fifteen days after inoculation, the mice were sacrificed and dissected to evaluate peritoneal dissemination, metastasis to the diaphragm and liver, and ascites formation. Omental tumors were paraffin-embedded, sectioned, and subjected to hematoxylin and eosin staining and immunohistochemistry. These experiments were approved by the Committee for Ethics of Animal Experimentation and were conducted in accordance with the guidelines for animal experiments of the National Cancer Center. Neither randomization nor blinding was used in this study.

### Glucose and lactate assay

Cells were washed with cold PBS and lysed in 0.1% Triton X-100. The lysates were filtered using an Amicon Ultra-3 kDa cutoff (Merck). Glucose and lactate levels were quantitated using a Glucose Assay kit-WST and Lactate Assay kit-WST (Dojindo).

### Statistical analysis

The data are representative of at least three independent experiments and presented as mean ± SD. Statistical analysis was performed using GraphPad Prism 8 (GraphPad Software). Statistical significance (defined as *P* < 0.05) was calculated using a two-tailed, unpaired Student’s *t*-test, one-way ANOVA and Tukey’s multiple comparison test, or Mann–Whitney test. Correlation analysis and calculation of Pearson’s correlation coefficient were also performed using GraphPad Prism 8. The sample size was not statistically predetermined but was chosen based on our experience and the standards in the fields.

## Results

### Identification of PLEKHA5 as a tyrosine-phosphorylated protein in DGC cells with Met gene amplification

We previously observed that the Met-inhibitor treatment of 58As9 DGC cells, which possess Met gene amplification and are addicted to Met signaling, greatly reduced overall protein tyrosine phosphorylation^21^. From this result, we hypothesized that most of the tyrosine-phosphorylated proteins in 58As9 cells are phosphorylated downstream of Met and may include critical mediators of Met signaling. To evaluate this hypothesis, we affinity-purified the tyrosine-phosphorylated proteins from 58As9 cells and systematically identified them by mass spectrometry. As listed in Supplementary Table 6, the identified proteins include the known downstream effectors of HGF/Met signaling such as HRS/HGS, PTPN11, and β-catenin, warranting their further examination as Met effectors. Among these, we focused on PLEKHA5, a member of the PLEKHA family of proteins, as this protein was poorly characterized.

### PLEKHA5 is tyrosine-phosphorylated downstream of Met

**Fig. 1:**
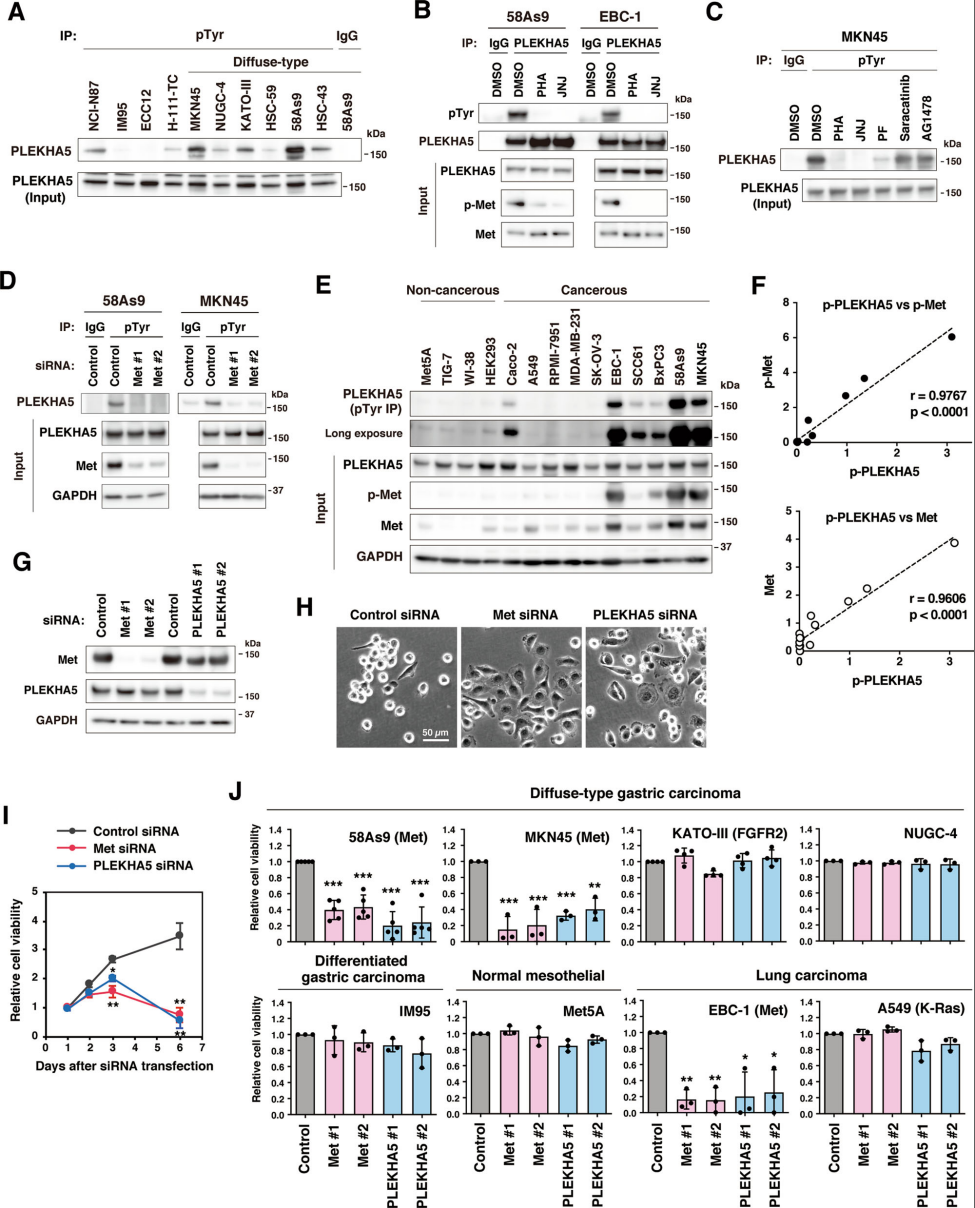
PLEKHA5 is tyrosine-phosphorylated downstream of Met and is required for the growth of Met-addicted carcinoma cells. **A** Gastric carcinoma cell lines were subjected to immunoprecipitation with an anti-phosphotyrosine (pTyr) antibody followed by immunoblotting with an anti-PLEKHA5 antibody. **B** 58As9 diffuse-type gastric carcinoma (DGC) cells and EBC-1 lung carcinoma cells were treated with Met inhibitors PHA-665752 (PHA) or JNJ-38877605 (JNJ) at 300 nM for 2 h and subjected to immunoprecipitation with anti-PLEKHA5 antibody and immunoblotting. **C** MKN45 DGC cells were treated with Met inhibitors, PHA, JNJ, or PF-2341066 (PF) at 300 nM, and the Src inhibitor saracatinib or EGFR inhibitor AG1478 at 10 µM for 2 h, and then subjected to immunoprecipitation and immunoblotting. **D** 58As9 and MKN45 cells were transfected with Met siRNAs and then subjected to immunoprecipitation and immunoblotting. **E** Tyrosine phosphorylation and expression of PLEKHA5 and Met in a panel of normal and cancerous cell lines were examined by immunoprecipitation and immunoblotting. **F** The correlation between phosphorylation and expression of PLEKHA5 and Met was examined by quantitative analysis of the immunoblotting data and evaluated with Pearson’s correlation coefficient. **G** 58As9 cells transfected with Met or PLEKHA5 siRNAs for 48 h were analyzed by immunoblotting. **H** Morphology of cells transfected with the indicated siRNAs. **I** Time-course of cell viability after Met or PLEKHA5 siRNA transfection in 58As9 cells. Bars, SD (*n* = 4). *P < 0.001; and **P < 0.0001. **J** Diffuse-type gastric carcinoma (58As9, MKN45, NUGC-4, KATO-III), intestinal-type gastric carcinoma (IM95), lung carcinoma (EBC-1 and A549), and normal mesothelial (Met5A) cell lines were transfected with Met or PLEKHA5 siRNAs and their viability was examined at 6 days after transfection. Of these, 58As9, MKN45, and EBC-1 cells have Met gene amplification. Bars, SD (*n* = 5 for 58As9, 4 for KATO-III, and 3 for others). *P < 0.01; **P < 0.005; and ***P < 0.0005.

We first examined the expression and tyrosine phosphorylation of PLEKHA5 in a panel of gastric cancer cell lines. Although PLEKHA5 expression was detected in all the cell lines tested, its tyrosine phosphorylation tended to be higher in diffuse-type gastric cancer cell lines, especially in MKN45 and 58As9 cells, both of which have Met gene amplification^21^ (Fig. 1A). Treatment of 58As9 and MKN45 cells with Met inhibitors PHA-665752, JNJ-38877605, and crizotinib (PF-2341066) markedly blocked the tyrosine phosphorylation of PLEKHA5 (Fig. 1B, C). Similarly, Met knockdown by siRNA transfection remarkably reduced the level of PLEKHA5 tyrosine phosphorylation in these cells (Fig. 1D). In contrast, treatment of the cells with an Src inhibitor (saracatinib) or an EGFR inhibitor (AG1478) did not have an obvious effect on the tyrosine phosphorylation of PLEKHA5 (Fig. 1C). PLEKHA5 was also tyrosine-phosphorylated in a Met activity-dependent manner in EBC-1 lung squamous cell carcinoma cells (Fig. 1B) that have Met gene amplification and are addicted to Met signaling^26^.

The correlation between PLEKHA5 and Met was further examined in a panel of non-cancerous and cancerous cell lines (Fig. 1E). Phosphorylation of Met and PLEKHA5 was barely detected in non-cancerous cell lines, whereas it was high in several cancerous cell lines. Notably, there was a strong correlation between PLEKHA5 phosphorylation and the expression and phosphorylation, i.e., activation status, of Met (Fig. 1F). Taken together, these results indicate that PLEKHA5 is tyrosine-phosphorylated downstream of Met signaling.

### PLEKHA5 is required for the growth of Met-addicted carcinoma cells

We then examined the effect of PLEKHA5 and Met knockdown on cell growth. Immunoblot analysis confirmed a successful reduction of Met and PLEKHA5 proteins by transfection with two different specific siRNAs (Fig. 1G). These knockdown cells exhibited a flattened morphology compared with the parental cells that had a rounded and refractile morphology (Fig. 1H). As expected, Met knockdown suppressed the growth of 58As9, which was evident 3 days after siRNA transfection (Fig. 1I, J). Likewise, PLEKHA5 knockdown significantly reduced cell growth (Fig. 1I, J). Met or PLEKHA5 silencing also blocked the growth of MKN45 cells but did not affect that of other gastric cancer cell lines, including KATO-III that has FGFR2 gene amplification, and normal mesothelial cell line without Met gene amplification (Fig. 1J). Similar to DGC cells, Met or PLEKHA5 knockdown significantly suppressed the growth of Met-addicted EBC-1 lung carcinoma cells (Fig. 1J). In contrast, it had little effect on the growth of A549 lung adenocarcinoma cells that have a K-Ras activating mutation (Fig. 1J). These results demonstrate that PLEKHA5 selectively regulates the growth of Met-addicted carcinoma cells.

By phosphoproteomic analysis of 58As9 cells, we identified PLEKHA6, another member of the PLEKHA family of proteins with unknown function (Supplementary Table 6). PLEKHA6 was also tyrosine-phosphorylated downstream of Met (Supplementary Fig. 1A). Nevertheless, the knockdown of PLEKHA6 had an insignificant effect on the growth of Met-addicted DGC cells (Supplementary Fig. 1B, C). Consequently, none of the PLEKHA family proteins but PLEKHA5 might regulate the growth of DGC cells.

### Gene signatures of PLEKHA5-silenced cells

**Fig. 2:**
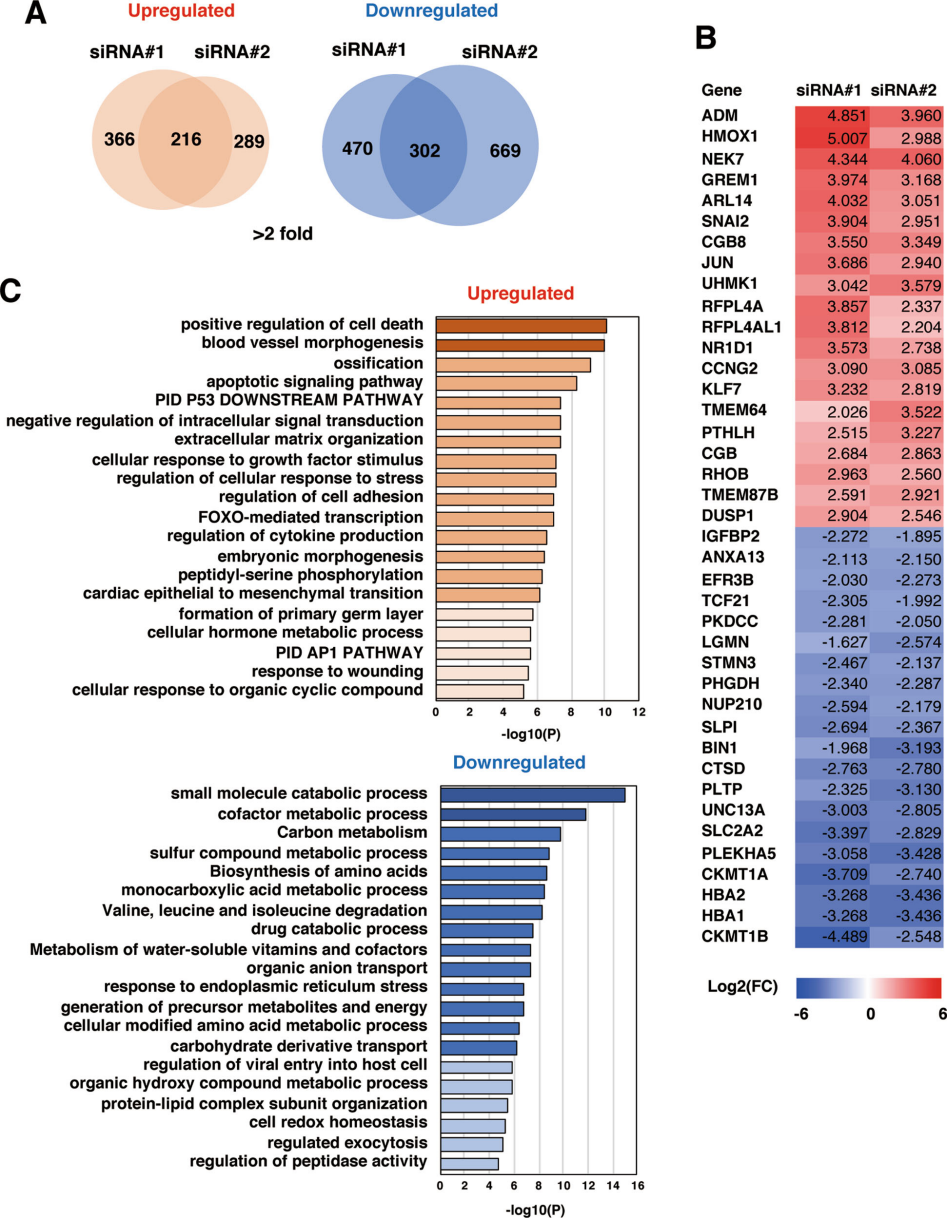
Gene expression analysis of PLEKHA5-silenced gastric carcinoma cells. **A** Venn diagrams of overlapping gene sets for commonly upregulated and downregulated genes in 58As9 cells transfected with two different PLEKHA5 siRNAs compared with the control cells. **B** Heat map of the top 20 commonly upregulated and downregulated genes in PLEKHA5 knockdown cells. The numbers are log2 fold changes compared to the control. **C** Enrichment analysis of the commonly upregulated and downregulated gene sets.

To gain insights into how PLEKHA5 regulates the growth of Met-addicted cells, changes in the gene expression profiles of 58As9 cells after silencing PLEKHA5 were determined by DNA microarray analysis. We identified 216 and 302 genes that were commonly upregulated and downregulated, respectively, in 58As9 cells treated with two different siRNAs compared with the control cells (Fig. 2A, B). The gene sets were further subjected to enrichment analysis to obtain their gene signatures (Fig. 2C). Interestingly, cell death- and apoptosis-related genes were significantly enriched in the upregulated gene set. In contrast, metabolism-related genes were significantly enriched in the downregulated gene set.

### PLEKHA5 regulates the survival of Met-addicted carcinoma cells

**Fig. 3:**
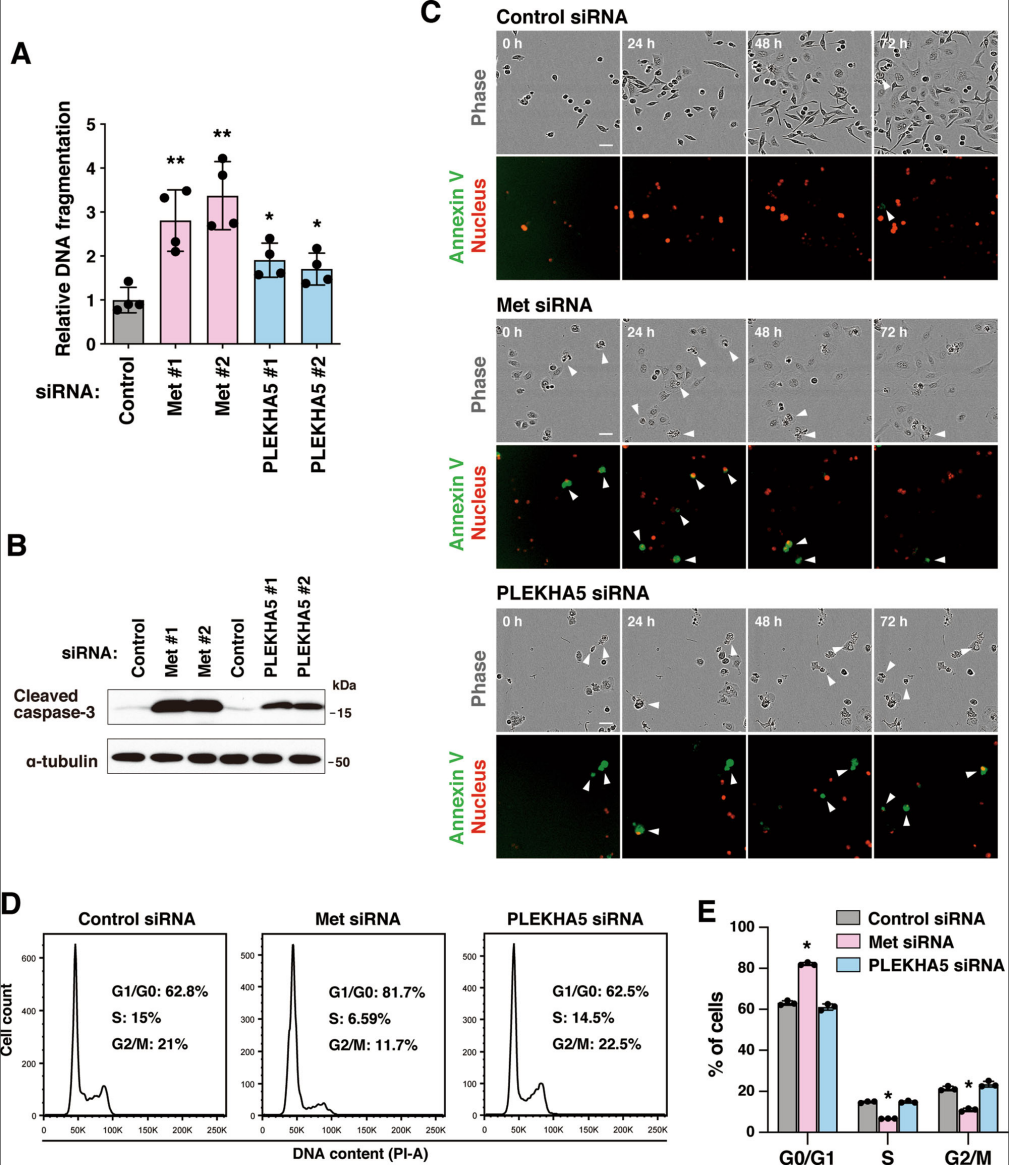
PLEKHA5 silencing induces apoptosis in Met-addicted DGC cells. **A** DNA fragmentation in 58As9 cells transfected with the indicated siRNAs. Bars, SD (*n* = 4). *P < 0.05; and **P < 0.01. **B** The amount of the pro-apoptotic protein, cleaved caspase-3, was examined by immunoblotting. **C** Image sequences of 58As9 cells fluorescently labeled for the apoptosis marker annexin V and nuclei were obtained by live-cell imaging. Arrowheads denote the cells undergoing apoptosis. Bars, 50 µm. **D** DNA content of 58As9 cells transfected with Met or PLEKHA5 siRNA was examined by propidium iodide staining and FACS analysis. **E** Quantification of cell populations in each cell cycle phase. Bars, SD (*n* = 3). *P < 0.0005.

Based on the DNA microarray results, we examined whether PLEKHA5 knockdown induces apoptotic cell death. Downregulation of Met or PLEKHA5 in 58As9 cells significantly induced DNA fragmentation (Fig. 3A) and increased the level of cleaved caspase-3, a pro-apoptotic protein (Fig. 3B). Similar results were obtained with MKN45 cells (Supplementary Fig. 2A). In addition, live-cell imaging demonstrated that silencing Met or PLEKHA5 clearly promoted the emergence of apoptotic cells that were labeled with annexin V (Fig. 3C and Supplementary Movies 1–3). These results demonstrate that PLEKHA5 is required for the survival of Met-addicted carcinoma cells.

As PLEKHA5 was reported to regulate cell cycle progression in melanoma cells^27^, the effect of PLEKHA5 knockdown on cell cycle progression was examined. Knockdown of Met significantly increased the population of cells in the G1 phase and decreased that of cells in the S and G2/M phases (Fig. 3D, E). In contrast, PLEKHA5 knockdown did not affect the population of cells in each cell cycle phase (Fig. 3D, E). Therefore, at least in DGC cells, PLEKHA5 is more likely to regulate the cell survival and apoptotic processes rather than cell cycle progression.

### PLEKHA5 knockdown suppresses the growth of DGC cells resistant to Met inhibitors

**Fig. 4:**
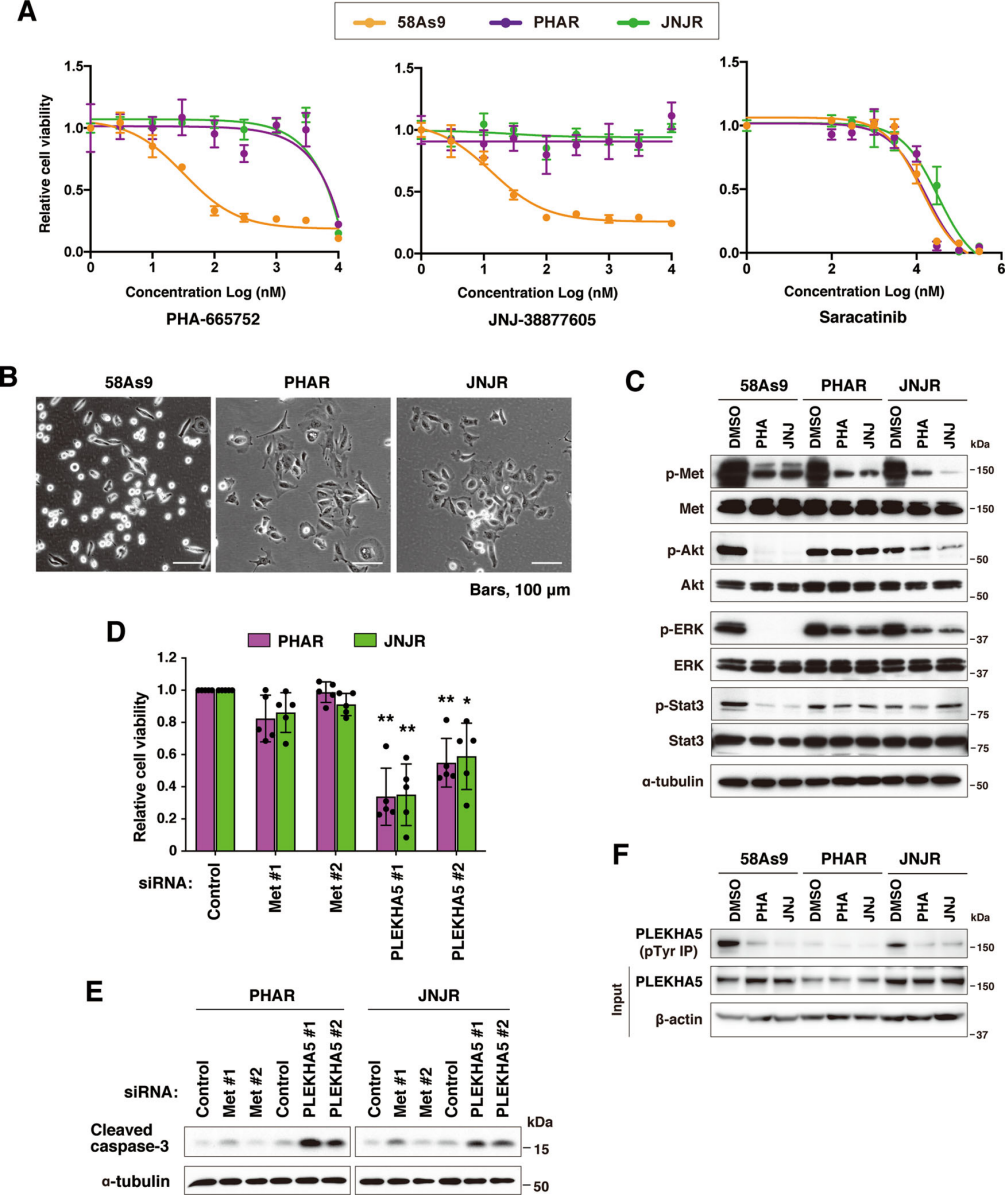
PLEKHA5 knockdown suppressed the growth of Met-inhibitor-resistant DGC cells. **A** Met-inhibitor-resistant 58As9 cells, PHAR and JNJR, were established by continuously culturing 58As9 cells in the presence of PHA-665752 and JNJ-38877605, respectively. The cells were then treated with increasing concentrations of Met inhibitors or an Src inhibitor, saracatinib, and their viability was examined. Bars, SD (*n* = 4). **B** Phase-contrast images show the morphology of 58As9, PHAR, and JNJR cells. **C** Immunoblot analysis of cells treated with PHA-665752 (PHA) and JNJ-38877605 (JNJ) at 300 nM for 2 h. **D** PHAR and JNJR cells were transfected with Met or PLEKHA5 siRNA and their viability was examined. Bars, SD (*n* = 5). *P < 0.05; and **P < 0.005. **E** The level of cleaved caspase-3 in PHAR and JNJR cells transfected with the indicated siRNAs was examined by immunoblotting. **F** Phosphorylation status of PLEKHA5 in PHAR and JNJR cells treated with Met inhibitors as in **C** was determined by immunoprecipitation and immunoblot analyses.

We next examined the requirement of PLEKHA5 for the growth of Met-inhibitor-resistant DGC cells. To this end, 58As9 cells were continuously cultured in the presence of Met inhibitors PHA-665752 or JNJ-38877605, and cells that acquired tolerance to Met inhibitors were obtained. PHA-665752 and JNJ-38877605 resistant cells were named as PHAR and JNJR, respectively. These cells exhibited marked resistance to Met inhibitors compared to the parental cells, whereas their sensitivity to an Src inhibitor, saracatinib, was unchanged (Fig. 4A). These resistant cells exhibited fibroblast-like and flattened morphology (Fig. 4B), similar to the Met knockdown cells (Fig. 1H, middle).

The activation status of Met and downstream signaling proteins was then examined by immunoblotting (Fig. 4C). Met phosphorylation in PHAR and JNJR cells was still decreased upon Met-inhibitor treatment. Further, we could not detect any mutations in the Met sequence of these cells, indicating that the resistance is not due to an activating mutation of Met. Although Met inhibitors markedly reduced the phosphorylation of Akt, ERK, and Stat3 in parental 58As9 cells, they were less effective in PHAR and JNJR cells.

As expected, the growth of these cells was no longer affected by Met silencing (Fig. 4D). In contrast, the knockdown of PLEKHA5 significantly suppressed the growth of these Met-inhibitor-resistant cells. Consistently, PLEKHA5 knockdown increased the level of cleaved caspase-3, whereas Met knockdown exhibited a minimum effect (Fig. 4E). These results suggest that Met-inhibitor-resistant cells are no longer addicted to Met, but still depend on PLEKHA5 to grow. Noticeably, basal levels of PLEKHA5 phosphorylation were reduced in the Met-inhibitor-resistant cells (Fig. 4F), indicating that PLEKHA5 exerts its pro-survival functions independently of Met in these cells.

### PLEKHA5 regulates the migration, invasion, and peritoneal dissemination of Met-addicted DGC cells

**Fig. 5:**
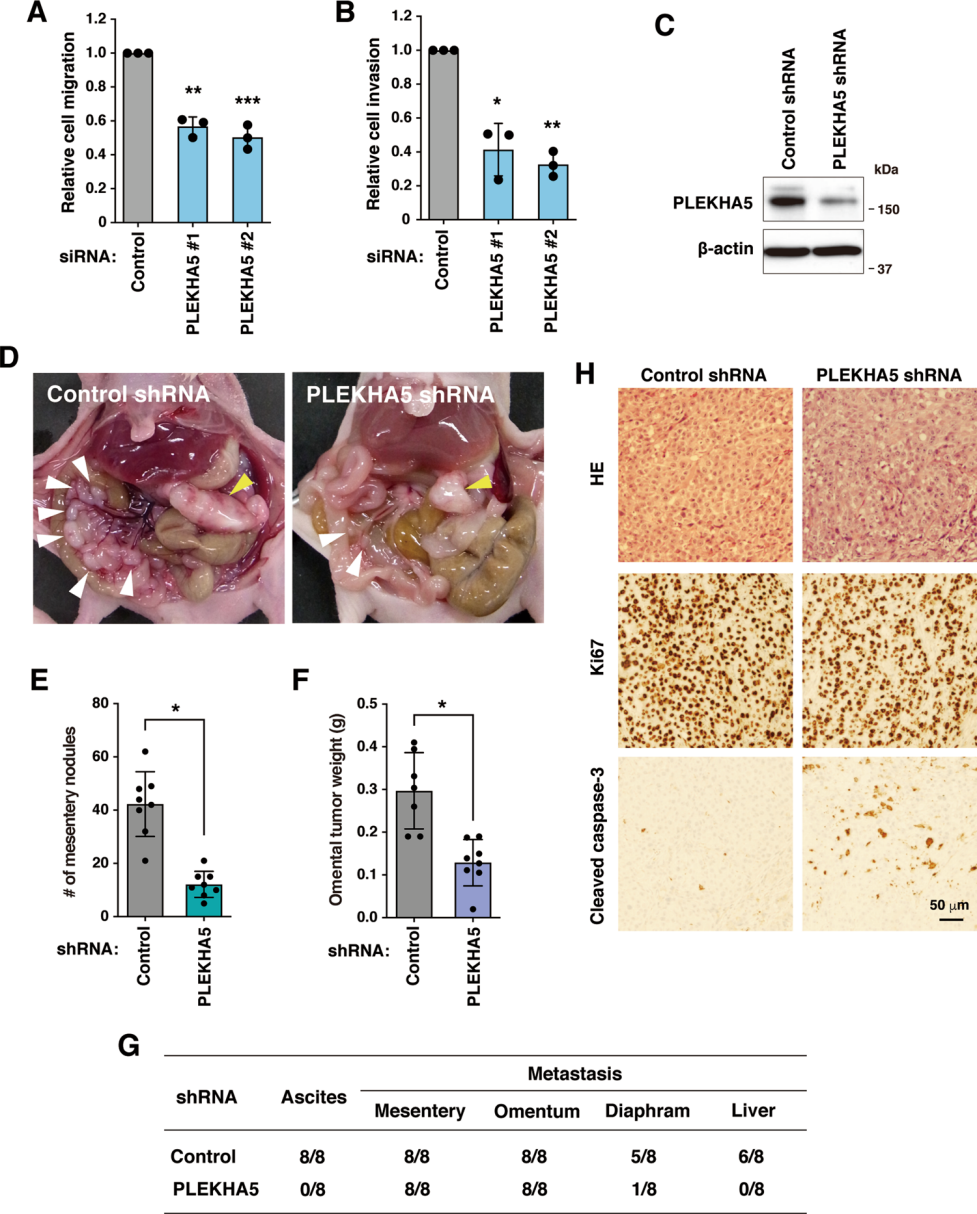
PLEKHA5 regulates the migration, invasion, and peritoneal dissemination of DGC cells. **A**, **B** 58As9 cells transfected with PLEKHA5 siRNA were analyzed for migration through Transwell inserts for 8 h (**A**) or for invasion through Matrigel-coated Transwell inserts for 24 h (**B**). Bars, SD (*n* = 3). *P < 0.001; **P < 0.0005; and ***P < 0.0001. **C** Cells infected with lentiviruses expressing control or PLEKHA5 shRNA were subjected to immunoblotting. **D** Cells expressing control or PLEKHA5 shRNA were intraperitoneally injected into nude mice. Representative macroscopic views of metastatic tumor nodules formed in the omentum (yellow arrowheads) and mesentery (white arrowheads) are shown. Reddish bloody ascites were seen in the control mice. **E** The number of mesenteric tumor nodules equal to or larger than 1 mm in diameter. Bars, SD (*n* = 8). *P < 0.001 by the Mann–Whitney test. **F** Omental tumor weight. Bars, SD (*n* = 7 for control and 8 for PLEKHA5 shRNA). *P < 0.001 by the Mann–Whitney test. **G** Number of mice bearing ascites or tumors at the indicated site per total number of mice bearing tumors. **H** Omental tumor sections were stained with hematoxylin and eosin and with anti-Ki67 and cleaved caspase-3 antibodies.

DGC is characterized by rapid infiltrative growth and peritoneal dissemination. Therefore, the possible involvement of PLEKHA5 in the malignant phenotypes of DGC cells was investigated. We first examined the role of PLEKHA5 in the migration and invasion of DGC cells using Transwell assays. To exclude a possibility that the suppression of cell growth by PLEKHA5 silencing affects cell migration and invasion, the assays were carried out during 1–2 days after siRNA transfection when cell viability was not significantly affected (Fig. 1I). Knockdown of PLEKHA5 significantly suppressed the ability of 58As9 cells to migrate through the Transwell membrane and to invade the reconstituted basement membrane (Fig. 5A, B).

Next, the role of PLEKHA5 in the peritoneal dissemination of DGC cells was investigated in a mouse xenograft model. For the in vivo experiments, 58As9 cells were infected with lentiviruses expressing shRNA against PLEKHA5 or control shRNA. Immunoblot analysis confirmed a successful reduction in the level of PLEKHA5 proteins by shRNA transduction (Fig. 5C). These cells were injected intraperitoneally into nude mice and their ability to disseminate to abdominal tissues was examined. Cells expressing PLEKHA5 shRNA showed a significant decrease in the formation of omental and mesenteric tumors as compared with control cells (Fig. 5D–F). In addition, PLEKHA5 knockdown markedly reduced the incidence of bloody ascites formation and tumor dissemination to the diaphragm and liver (Fig. 5G). Histological analysis showed that the omental tumors with PLEKHA5 knockdown contained more apoptotic cells positive for cleaved caspase-3 compared to control tumors, whereas the number of Ki67 positive growing cells showed no obvious change (Fig. 5H). Taken together, our results indicate that PLEKHA5 is a key regulator of malignant phenotype acquisition and progression of Met-addicted DGC cells.

### PLEKHA5 regulates glycolytic metabolism and the JNK pathway

**Fig. 6:**
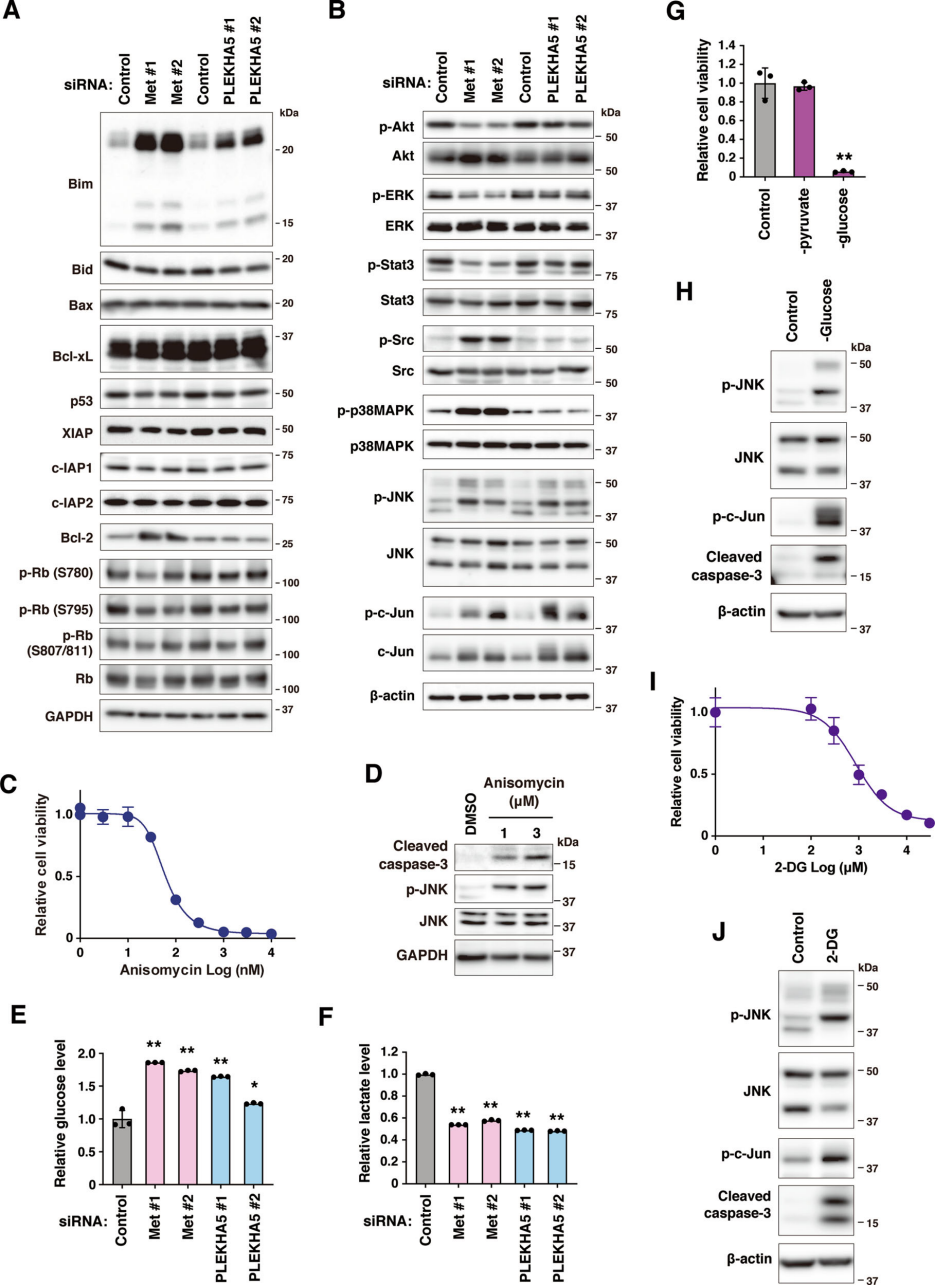
PLEKHA5 knockdown dysregulates glycolysis and induces JNK activation. **A**, **B** 58As9 cells transfected with Met and PLEKHA5 siRNAs were subjected to immunoblot analysis with the indicated antibodies against proteins involved in apoptosis and cell cycle regulation (**A**) or intracellular signaling (**B**). **C** Cells were treated with increasing concentrations of anisomycin for 3 days and their viability was examined. **D** Cells treated with anisomycin were analyzed by immunoblotting with the indicated antibodies. **E**, **F** Cellular levels of glucose (**E**) and lactate (**F**) were quantified in 58As9 cells transfected with Met and PLEKHA5 siRNAs as described in the “Materials and methods” section. **G** Viability of 58As9 cells starved for pyruvate or glucose for 3 days. **H** Cells starved for glucose were subjected to immunoblotting with the indicated antibodies. **I** Viability of 58As9 cells treated with increasing concentrations of 2-deoxyglucose (2-DG) for 3 days. **J** Cells treated with 10 mM 2-DG for 1 day were subjected to immunoblotting with the indicated antibodies. Bars, SD (*n* = 3). *P < 0.005; and **P < 0.0001.

To understand the cellular function of PLEKHA5, we determined the changes in the expression and activation status of apoptosis- and cell cycle-related molecules upon PLEKHA5 knockdown in 58As9 cells (Fig. 6A). Among the pro-apoptotic proteins examined, Bim expression was increased in both Met- and PLEKHA5-silenced cells. Similar results were seen in MKN45 cells (Supplementary Fig. 2A). The antiapoptotic protein Bcl-2 was increased in Met but not in PLEKHA5 knockdown cells. No obvious changes were observed in p53, IAP family, or other Bcl family proteins. This observation was inconsistent with the microarray data in which p53 pathway genes were upregulated upon PLEKHA5 knockdown (Fig. 2C). We found that p53 expressed in 58As9 cells contained an inactivating mutation and indeed failed to induce p21 accumulation upon DNA damage or MDM2 inhibition (Supplementary Fig. 3A, B). In addition, we noticed that the p53 pathway gene set largely overlapped with other apoptosis-related gene sets upregulated in PLEKHA5 knockdown cells (Supplementary Table 4). Taken together, PLEKHA5 knockdown seems to upregulate pro-apoptotic gene sets, including p53 pathway genes, independently of p53 activity. In melanoma cells, PLEKHA5 knockdown was reported to reduce RB phosphorylation^27^. However, RB phosphorylation was unchanged upon Met or PLEKHA5 silencing in this cell type.

The activation status of signaling molecules was then examined (Fig. 6B). As expected, Met knockdown suppressed the phosphorylation of Akt, ERK, and Stat3, but increased that of Src. In contrast, PLEKHA5 knockdown did not affect these signaling molecules. Met silencing increased the phosphorylation of p38MAPK and JNK, both of which mediate stress responses and have pro-apoptotic functions^28^. Phosphorylation of JNK, but not p38MAPK, was also increased by PLEKHA5 knockdown. Phosphorylation and expression of c-Jun, a target of JNK, were increased in both Met and PLEKHA5-silenced cells. In line with these observations, DNA microarray analysis revealed that c-Jun, as well as stress response and AP1 pathway genes, were upregulated in PLEKHA5-silenced cells (Fig. 2B, C). Additionally, JNK was previously shown to stabilize and activate Bim^29^. These observations indicate that PLEKHA5 knockdown results in JNK activation, which in turn induces apoptotic cell death. Supporting this idea, treatment of 58As9 cells with anisomycin, an activator of JNK, markedly decreased their cell viability in a dose-dependent manner (Fig. 6C) and induced apoptotic cell death as revealed by an increase in cleaved caspase-3 levels (Fig. 6D).

Inhibition of glycolytic metabolism induces apoptosis via JNK activation in cancer cells relying on glycolysis^30^. Our DNA microarray data showed that genes involved in a variety of metabolic processes were downregulated in PLEKHA5-silenced cells (Fig. 2C). Therefore, we examined the possible involvement of PLEKHA5 in glycolysis by measuring the cellular content of glucose and lactate, the source and the main product of glycolysis, respectively. The cellular level of lactate was significantly reduced, whereas that of glucose was increased in Met and PLEKHA5 knockdown cells (Fig. 6E, F). Similar tendencies were also observed in Met-addicted MKN45 cells but not in NUGC-4 cells (Supplementary Fig. 2B). Deprivation of glucose, but not pyruvate, another major product of glycolysis, from culture media resulted in markedly reduced cell viability (Fig. 6G), which was associated with JNK activation and induction of apoptosis, as evidenced by increased caspase-3 cleavage (Fig. 6H). Likewise, treatment of cells with 2-deoxyglucose (2-DG), an inhibitor of glycolysis, decreased cell viability in a dose-dependent manner (Fig. 6I), and provoked JNK activation and apoptosis (Fig. 6J). Taken together, these results suggest that PLEKHA5 knockdown impairs glycolysis, leading to JNK activation and apoptotic cell death.

## Discussion

Met gene amplification has been associated with malignant progression, drug resistance, and poor prognosis in a subset of carcinomas^16,31^. Accordingly, Met inhibitors are being currently evaluated in clinical trials; however, their efficacy has been disappointing^32^. This is partially due to the lack of appropriate biomarkers for the detection of Met-addicted carcinoma. In this study, we identified PLEKHA5 as a tyrosine-phosphorylated protein in DGC cells with Met gene amplification that were addicted to Met. We showed that the tyrosine phosphorylation of PLEKHA5 was Met-dependent and correlated with the expression and phosphorylation of Met. Thus, PLEKHA5 phosphorylation could be a biomarker for Met-addicted carcinoma. In addition, PLEKHA5 silencing selectively suppressed the growth of carcinoma cells with Met gene amplification. Importantly, this effect was observed even though the carcinoma cells acquired resistance to Met inhibitors. Considering that PLEKHA5 silencing had a negligible effect on the growth of normal cells, blocking PLEKHA5 function may have therapeutic potential against Met-addicted carcinomas with minimal side effects. The mechanism by which PLEKHA5 phosphorylation occurs downstream of Met remains obscure. The detailed analysis of the mechanism and importance of PLEKHA5 phosphorylation in Met signaling are critical for evaluating PLEKHA5 as a therapeutic target and a biomarker for Met-addicted carcinoma.

DGC is characterized by rapid invasive growth and frequent peritoneal dissemination. The process of peritoneal dissemination includes attachment of DGC cells to the mesothelium, invasion into the submesothelial connective tissue, and subsequent growth to form tumors^33,34^. Our results demonstrate that PLEKHA5 silencing markedly blocked the peritoneal dissemination of DGC cells. This inhibitory effect was most probably achieved by reduced cell migration, invasion, and survival due to PLEKHA5 silencing. Consistent with our findings, a previous study demonstrated that PLEKHA5 is required for the growth and invasion of melanoma cells that are prone to brain metastasis and their transmigration through the blood–brain barrier^35^. Although the activation and genetic status of Met in the melanoma cells used in the study are unknown, Met gene alterations are rare in melanoma^36^. Considering that PLEKHA5 is widely expressed in cancer cell lines as shown previously^37^ and in this study, PLEKHA5 may also act downstream of oncogenic molecules besides Met and may have other cellular functions in some contexts.

Liu et al.^38^ recently reported that PLEKHA5 acts as a tumor suppressor in breast cancer metastasis, where PLEKHA5 knockout promoted migration and invasion. This apparently contradicts the tumor-promoting functions of PLEKHA5 reported in this study and previous studies in melanoma described above^27,35^. At present, the precise reason for this discrepancy is unclear. However, it is possible that PLEKHA5 has both tumor-promoting and suppressive functions, depending on the cell context and cancer types. As discussed by Lie et al.^38^, it is also possible that splicing isoforms of PLEKHA5, which were described in a previous study^24^, have opposing functions in tumor formation and metastasis. Further characterization of the splicing isoforms is definitely important not only to address this issue but also to prevent potential adverse effects when targeting PLEKHA5.

Cell growth is the sum of fine-tuned regulation of cell cycle progression and cell survival. We observed that Met knockdown in Met-addicted DGC cells caused cell cycle arrest in addition to inducing apoptosis. In contrast, PLEKHA5 knockdown reduced cell survival by inducing apoptosis without affecting cell cycle progression. Met regulates cell growth by activating the major downstream pathways mediated by PI3-kinase/Akt, Ras/ERK, and Stat3^13^. PLEKHA5 was suggested to regulate PI3-kinase signaling^39^, as this molecule possesses a PH domain and associates with phosphoinositides. Although PLEKHA5 was reported to preferentially bind to PI(3)P, PI(4)P, PI(5)P, and PI(3,5)P_2_^24^, these phosphoinositides mainly have roles in membrane trafficking and contribute little to PI3-kinase/Akt signaling in which PI(3,4)P_2_ and PI(3,4,5)P_3_ are mediators^40,41^. Indeed, we showed that PLEKHA5 silencing did not significantly affect the PI3-kinase/Akt signaling and the Ras/ERK and Stat3 pathways. Instead, downregulation of PLEKHA5 induced activation of JNK, which has pro-apoptotic functions^42^. Thus, these findings suggest that cell cycle progression by Met is mediated by major signaling pathways, in which PLEKHA5 is not involved, whereas cell survival is controlled by PLEKHA5-dependent regulation of JNK. Further studies are thus required to elucidate the precise cellular functions of PLEKHA5.

Cancer cells rely on aerobic glycolysis to maintain rapid growth and to escape apoptosis in a process known as the Warburg effect. Our results suggest that PLEKHA5 is required for the proper glycolytic process in Met-addicted DGC cells. In these cells, glucose deprivation or inhibition of glycolysis induced JNK activation and apoptotic cell death. Therefore, the JNK activation observed in PLEKHA5-silenced cells seems to be secondarily caused by an impaired glycolytic process. It is currently unclear how PLEKHA5 regulates the glycolytic process. A recent study demonstrated that enhanced glycolysis by oncogenic EGFR supports the survival of lung cancer cells by inhibiting EGFR degradation, and that impairment of this system leads to apoptosis via JNK activation^30^. Accordingly, future studies should explore whether amplification of Met elicits similar metabolic and cell survival systems in DGC and whether PLEKHA5 mediates this process downstream of Met.

In conclusion, our results demonstrate that PLEKHA5 regulates survival and malignant phenotypes, including peritoneal dissemination, of DGC cells with Met gene amplification. These results raise a possibility that PLEKHA5 is a novel mediator of oncogenic Met signaling. Obviously, more detailed mechanistic analyses are required to understand the precise relationship between PLEKHA5 and Met. Our findings may provide a novel approach for molecular targeted therapy targeting PLEKHA5 in patients with Met-addicted carcinoma.

## Supplementary information

Movie 1

Movie 2

Movie 3

Supplementary figures and legends

Supplementary Table 1

Supplementary Table 2

Supplementary Table 3

Supplementary Table 4

Supplementary Table 5

Supplementary Table 6
