# Supplementary material for: PLEKHA5 regulates the survival and peritoneal dissemination of diffuse-type gastric carcinoma cells with Met gene amplification
Source: Oncogenesis. 2021 Mar 6;10(3):25. doi: 10.1038/s41389-021-00314-1 (PMC7936979; doi:10.1038/s41389-021-00314-1)
Supplement: Supplementary file 4 — Supplementary figures and legends [file 41389_2021_314_MOESM4_ESM.docx]

**Supplementary figures and figure legends**

*Supplementary Fig 1. PLEKHA6 is tyrosine-phosphorylated downstream of Met but not required for the growth of Met-addicted gastric carcinoma cells*

A, 58As9 cells were treated with DMSO or Met inhibitors PHA-665752 (PHA) and JNJ-388760 (JNJ) and subjected to immunoprecipitation with anti-phosphotyrosine (pTyr) antibody. The immunoprecipitates were then analyzed by immunoblotting with anti-PLEKHA6 antibody. B, Cells were transfected with control or PLEKHA6 siRNA and subjected to immunoblotting with indicated antibodies. C, Viability of cells transfected with control or PLEKHA6 siRNA. Bars, SD (*n* = 3).

**

*Supplementary Fig 2. Apoptosis and glycolytic changes were induced by PLEKHA5 knockdown in MKN45 cells*

A, MKN45 cells were transfected with Met and PLEKHA5 siRNAs, and subjected to immunoblot analysis. B, Cellular levels of glucose and lactate were quantified in MKN45 and NUGC-4 cells transfected with Met and PLEKHA5 siRNAs. Bars, SD (*n* = 3). *, *P* < 0.05; and **, *P* < 0.0001.

*
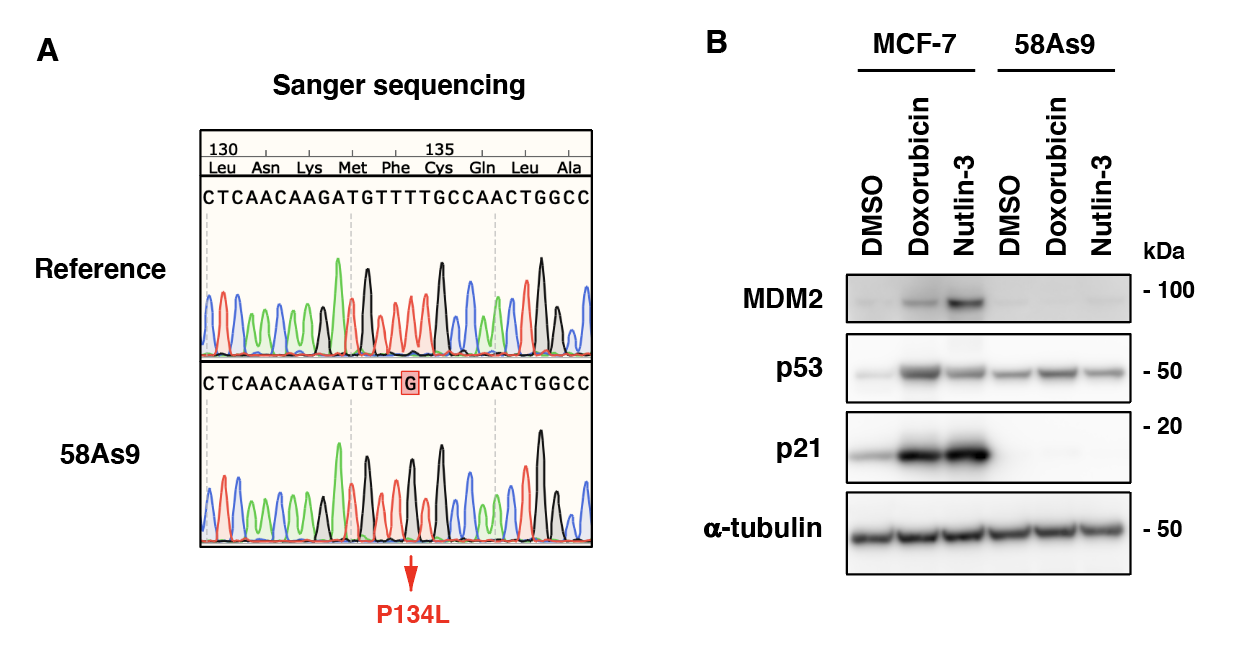
*

*Supplementary Fig 3. p53 is inactivated by P134L mutation in 58As9 cells*

A, DNA sequence chromatograms showing that p53 expressed in 58As9 cells has the P134L inactivating mutation. B, MCF-7 and 58A9 cells were treated with doxorubicin or nutlin-3, both of which induce p53 activation, and subjected to immunoblotting. Doxorubicin and nutlin-3 cause DNA damage and MDM2 inhibition, respectively. Doxorubicin or nutlin-3 treatment induces accumulation of MDM2, p53, and p21 in MCF-7 cells but not in 58As9 cells.

*Movie 1. Live cell imaging of 58As9 cells transfected with control siRNA*

58As9 cells were transfected with control siRNA for 2 days and then observed by live cell imaging as described in Materials and Methods. Phase contrast and fluorescent images were shown. Green, the cells undergoing apoptosis as detected by Annexin V. Red, nuclei. Play rate, 10 fps. 1 h per frame, total 72 hours.

*Movie 2. Live cell imaging of 58As9 cells transfected with Met siRNA*

58As9 cells transfected with Met siRNA were observed as in Movie 1.

*Movie 3. Live cell imaging of 58As9 cells transfected with PLEKHA5 siRNA*

58As9 cells transfected with PLEKHA5 siRNA were observed as in Movie 1.
