# Supplementary material for: PLEKHA5 regulates the survival and peritoneal dissemination of diffuse-type gastric carcinoma cells with Met gene amplification
Source: Oncogenesis. 2021 Mar 6;10(3):25. doi: 10.1038/s41389-021-00314-1 (PMC7936979; doi:10.1038/s41389-021-00314-1)
Supplement: Supplementary file 5 — Supplementary Table 1 [file 41389_2021_314_MOESM5_ESM.docx]

**Supplementary Table 1. Human cell lines used in this study**

| Cell line | Cell type | Source | ID or references |
| --- | --- | --- | --- |
| NCI-N87 | Gastric cancer | ATCC | CRL-5822 |
| IM95 | Gastric cancer | JCRB | JCRB1075.0 |
| MKN45 | Gastric cancer | JCRB | JCRB0254 |
| NUGC-4 | Gastric cancer | JCRB | JCRB0834 |
| KATO-III | Gastric cancer | JCRB | JCRB0611 |
| HSC-59 | Gastric cancer | Dr Kazuyoshi Yanagihara | Ref. 1 |
| 58As9 | Gastric cancer | Dr Kazuyoshi Yanagihara | Ref. 2 |
| HSC-43 | Gastric cancer | Dr Kazuyoshi Yanagihara | Ref. 3 |
| ECC12 | Gastric cancer | RIKEN BRC | RCB1009 |
| H-111-TC | Gastric cancer | RIKEN BRC | RCB1884 |
| BxPC3 | Pancreatic cancer | ATCC | CRL-1687 |
| MDA-MB-231 | Breast cancer | ATCC | HTB-26 |
| RPMI-7951 | Melanoma | ATCC | HTB-66 |
| SK-OV-3 | Ovarian cancer | ATCC | HTB-77 |
| A549 | Lung cancer | ATCC | CCL-185 |
| Met5A | Mesothelial | ATCC | CRL-9444 |
| HEK293 | Embryonic kidney | JCRB | JCRB9068 |
| EBC-1 | Lung cancer | JCRB | JCRB0820 |
| WI-38 | Fibroblast | JCRB | IFO50075 |
| TIG-7 | Fibroblast | JCRB | JCRB0512 |
| Caco-2 | Colon cancer | DS Pharma Biomedical | DSPOCA001-ZS |
| SCC61 | Head and neck squamous cell carcinoma | Dr Alissa Weaver | Ref. 4 |
| MCF-7 | Breast cancer | Resource Center for Biomedical Research, Institute of Development, Aging and Cancer Tohoku University | TKG-0479 |

References

1. Yanagihara, K. et al. Establishment of two cell lines from human gastric scirrhous carcinoma that possess the potential to metastasize spontaneously in nude mice. *Cancer Sci.* **95**, 575-582 (2004).

2. Yanagihara, K. et al. Development and biological analysis of peritoneal metastasis mouse models for human scirrhous stomach cancer. *Cancer Sci.* **96**, 323-332 (2005).

3. Yanagihara, K., Kamada, N., Tsumuraya, M., Amano, F. Establishment and characterization of a human gastric scirrhous carcinoma cell line in serum-free chemically defined medium. *Int. J. Cancer* **54**, 200-207 (1993).

4. Clark, E. S., Whigham, A. S., Yarbrough, W. G., Weaver, A. M. Cortactin is an essential regulator of matrix metalloproteinase secretion and extracellular matrix degradation in invadopodia. *Cancer Res.* **67**, 4227-4235 (2007).
