# Supplementary material for: PLEKHA5 regulates the survival and peritoneal dissemination of diffuse-type gastric carcinoma cells with Met gene amplification
Source: Oncogenesis. 2021 Mar 6;10(3):25. doi: 10.1038/s41389-021-00314-1 (PMC7936979; doi:10.1038/s41389-021-00314-1)
Supplement: Supplementary file 6 — Supplementary Table 2 [file 41389_2021_314_MOESM6_ESM.docx]

**Supplementary Table 2. Commercially available antibodies used in this study**

| Antibody | Vendor | Catalog number |
| --- | --- | --- |
| Met | Santa Cruz Biotechnology | SC-10 |
| Met | Cell Signaling Technology | #3127 |
| p-Met (Y1234/1235) | Cell Signaling Technology | #3077 |
| Src | Cell Signaling Technology | #2109 |
| p-Src (Y416) | Cell Signaling Technology | #2101 |
| ERK | Cell Signaling Technology | #9102 |
| p-ERK (T202/Y204) | Cell Signaling Technology | #9101 |
| Akt | Cell Signaling Technology | #4691 |
| p-Akt (S473) | Cell Signaling Technology | #4060 |
| Stat3 | Santa Cruz Biotechnology | SC-482 |
| p-Stat3 (Y705) | Cell Signaling Technology | #9145 |
| p38MAPK | Cell Signaling Technology | #8690 |
| p-p38MAPK (T180/Y182) | Cell Signaling Technology | #4511 |
| SAPK/JNK | Cell Signaling Technology | #9252 |
| p-SAPK/JNK (T183/Y185) | Cell Signaling Technology | #4668 |
| c-Jun | Cell Signaling Technology | #9165 |
| p-c-Jun (S73) | Cell Signaling Technology | #3270 |
| Cleaved Caspase-3 | Cell Signaling Technology | #9664 |
| Bim | Cell Signaling Technology | #2933 |
| Bid | Cell Signaling Technology | #2002 |
| Bcl-2 | Cell Signaling Technology | #2870 |
| Bax | Cell Signaling Technology | #2772 |
| Bcl-xL | Cell Signaling Technology | #2764 |
| p53 | Cell Signaling Technology | #9282 |
| p53 | Santa Cruz Biotechnology | SC-126 |
| p21 | Cell Signaling Technology | #2947 |
| XIAP | Cell Signaling Technology | #2045 |
| c-IAP1 | Cell Signaling Technology | #7065 |
| c-IAP2 | Cell Signaling Technology | #3130 |
| Rb | Cell Signaling Technology | #9309 |
| p-Rb (S780) | Cell Signaling Technology | #8180 |
| p-Rb (S795) | Cell Signaling Technology | #9301 |
| p-Rb (S807/811) | Cell Signaling Technology | #8516 |
| MDM2 | Calbiochem | OP46 |
| GAPDH HRP | Cell Signaling Technology | #3683 |
| β-actin HRP | Cell Signaling Technology | #5125 |
| β -actin | Sigma | A2228 |
| α -tubulin | Sigma | T5168 |
| PLEKHA5 | Santa Cruz Biotechnology | SC-390311 |
| 4G10 | Merck | 05-321 |
| PLEKHA6 | Sigma | HPA028152 |
