# Supplementary material for: PLEKHA5 regulates the survival and peritoneal dissemination of diffuse-type gastric carcinoma cells with Met gene amplification
Source: Oncogenesis. 2021 Mar 6;10(3):25. doi: 10.1038/s41389-021-00314-1 (PMC7936979; doi:10.1038/s41389-021-00314-1)
Supplement: Supplementary file 10 — Supplementary Table 6 [file 41389_2021_314_MOESM10_ESM.docx]

**Supplementary Table 6. Proteins isolated from 58As9 cells by affinity purification of tyrosine-phosphorylated proteins**

| Function | Protein name |
| --- | --- |
| Cell-cell adhesion | Catenin δ-1, Catenin β-1, Plakophilin-3, ZO-2, Plakoglobin, Afadin, ZO-1 |
| Cell-extracellular matrix adhesion | Integrin-β4 |
| Cytoskeleton | Filamin-B, Actinin-4, Ezrin, p130Cas, ABLIM1, Myosin-Ie, Cortactin, CAPG, PDLIM5, DBNL, Septin-2 |
| Signal transduction | Met, EPS8, ARHG5, LAP2/Erbin, EGFR, KIRR1/KIRREL, Annexin A11, LRP6, Cytohesin-1, PTPRJ, Src, PTPN11, STAM2, Cbl, Shc1 |
| Membrane trafficking | BICD2, Flotillin-1, Sec24B, SCAM3, Src23A, Cytohesin-1, HRS/HGS |
| Stress response | Grp78, HSP7c, HSP71, TCPA, HSP76 |
| Redox | SQRD |
| Metabolism | 6-phosphofructokinase C, Pyruvate kinase isozymes M1/M2, ATP synthase subunit a, Aspartyl-tRNA synthetase, G6PDH, Aldo-keto reductase family 1 member C3 |
| Transcription &Translation | DDX3X, Nucleolin, HNRPF, IF4A1, EIF3L, EFTU, HNRPQ, YBX-1, HNRPK |
| DNA repair | Ku70/XRCC6, RuvBL2 |
| Unknown | PLEKHA5, PLEKHA6 |
